# *Advances in Respiratory Medicine*—Aims and Scopes Update

**DOI:** 10.3390/arm92020017

**Published:** 2024-03-19

**Authors:** Krzysztof Kuziemski, Monika Franczuk, Sebastian Majewski, Tadeusz M. Zielonka, Adam Barczyk

**Affiliations:** 1 Department of Pulmonology and Allergology, Faculty of Medicine, Medical University of Gdansk, 80-210 Gdansk, Poland; k.kuziemski@gumed.edu.pl; 2 Respiratory Physiopathology Department, National Tuberculosis and Lung Diseases Research Institute, 01-138 Warsaw, Poland; monika.franczuk@gmail.com; 3 Department of Pneumology, Medical University of Lodz, 90-419 Lodz, Poland; sebastian.majewski@umed.lodz.pl; 4 Department of Family Medicine, Faculty of Medicine, Medical University of Warsaw, 02-091 Warsaw, Poland; tmzielonka@wp.pl; 5 Department of Pneumonology, School of Medicine in Katowice, Medical University of Silesia in Katowice, 40-055 Katowice, Poland

*Advances in Respiratory Medicine*, which has been published by MDPI since 2022, serves as a platform for hosting pneumological studies [1]. The journal achieved its first Impact Factor of 1.8 in June 2023. In order to provide readers and potential authors with a more detailed and accurate understanding, we believe it is beneficial to update the Aims and Scope of the journal.

## 1. Aims

*Advances in Respiratory Medicine*, which started publishing in 1909, is a peer-reviewed, open access, bi-monthly journal of the Polish Respiratory Society [2]. It publishes research articles, review articles, systematic reviews, guidelines, editorials, short communications, etc. *Advances in Respiratory Medicine* publishes both clinical and basic research, and it is also open to the publication of phased trial work.

We aim to collect high-quality and cutting-edge articles of interest in respiratory medicine and provide an efficient, reliable, and guaranteed platform for scholars and readers. Full experimental and/or methodological details must be provided for research articles. Therefore, there is no restriction on the maximum length of papers or the number of electronic multimedia and supplementary files.

## 2. Scope

*Advances in Respiratory Medicine* is a journal of respiratory medicine, covering allergology, immunology, oncology, and infectious diseases of the respiratory system. It welcomes manuscripts related to respiratory system structure, physiology, pathology, diagnostics, and treatment. It includes, but is not limited to, manuscripts on the following subjects:

- Respiratory structure and physiopathology (including basic science, molecular aspects of pathology, mechanisms of lung injury and repair, immunology of the respiratory system, etc.);
- Clinical problems, rehabilitation, chronic care, and respiratory medicine (in general practice and primary care);
- Chronic airway diseases (asthma, COPD, bronchiectasis, chronic cough, etc.);
- Infectious diseases of the respiratory system (including tuberculosis and non-tuberculosis mycobacterial diseases, COVID-19, AIDS, and diseases caused by bacteria, viruses, and parasites);
- Interstitial lung diseases (all aspects);
- Genetic diseases with respiratory manifestations (cystic fibrosis, A1AT deficiency, etc.);
- Pulmonary vascular diseases (vasculitis, pulmonary embolism, pulmonary hypertension, etc.);
- Rare (orphan) diseases with respiratory system involvement;
- Respiratory intensive care (acute critical care, noninvasive ventilatory support, oxygen therapy, etc.);
- Thoracic oncology (lung cancer, pleural and mediastinal malignancies, etc.);
- Thoracic surgery and lung transplantation;
- Sleep-disordered breathing;
- Epidemiology and environment (occupational respiratory diseases, environmental exposure, tobacco, smoking control, etc.);
- Respiratory diagnostics (imaging, ultrasound, lung function testing, exercise testing, microbiology, endoscopic methods, etc.);
- Respiratory medicine in pediatrics (respiratory physiology and sleep, pediatric asthma and allergies, cystic fibrosis, respiratory infections, intensive care, epidemiology, diagnostics, etc.).
